# Causes of death after fluid bolus resuscitation: new insights from FEAST

**DOI:** 10.1186/1741-7015-11-67

**Published:** 2013-03-14

**Authors:** John Myburgh, Simon Finfer

**Affiliations:** 1 St George Clinical School, University of New South Wales, The George Institute for Global Health, L13, 321 Kent Street, Sydney 2000, Australia; 2 Royal North Shore Hospital, University of Sydney, The George Institute for Global Health, L13, 321 Kent Street, Sydney 2000, Australia

**Keywords:** albumin, cardiogenic shock, FEAST trial, fluid resuscitation, malaria, pediatrics, reperfusion injury, saline, sepsis, shock

## Abstract

The Fluid Expansion as Supportive Therapy (FEAST study) was an extremely well conducted study that gave unexpected results. The investigators had reported that febrile children with impaired perfusion treated in low-income countries without access to intensive care are more likely to die if they receive bolus resuscitation with albumin or saline compared with no bolus resuscitation at all. In a secondary analysis of the trial, published in *BMC Medicine*, the authors found that increased mortality was evident in patients who presented with clinical features of severe shock in isolation or in conjunction with features of respiratory or neurological failure. The cause of excess deaths was primarily refractory shock and not fluid overload. These features are consistent with a potential cardiotoxic or ischemia-reperfusion injury following resuscitation with boluses of intravenous fluid. Although these effects may have been amplified by the absence of invasive monitoring, mechanical ventilation or vasopressors, the results provide compelling insights into the effects of intravenous fluid resuscitation and potential adverse effects that extend beyond the initial resuscitation period. These data add to the increasing body of literature about the safety and efficacy of intravenous resuscitation fluids, which may be applicable to management of other populations of critically ill patients.

## Background

The results of the Fluid Expansion as Supportive Therapy (FEAST) trial [1] challenge many established principles about using intravenous fluids for resuscitation. Conducted in a pediatric population with compensated shock under conditions of extreme deprivation, the study found that bolus fluid resuscitation with albumin or saline was associated with a statistically significant increase in mortality at four and 48 hours compared with no boluses of fluid.

These compelling results question well-established recommendations for fluid boluses as a first-line intervention in hemodynamic resuscitation, not only in pediatric patients but also in all critically ill patients.

Debates and commentaries have questioned the generalizability of these results in high-income countries where invasive monitoring, mechanical ventilation and vasopressors are standards of care [2]. Concerns have also been expressed about the high proportion of patients with severe malaria and anemia in the FEAST study and whether the increased mortality was overly influenced by this cohort of patients [3].

Interpreting these results in disparate populations therefore requires further insights into the potential mechanisms of increased death associated with bolus resuscitation. The results presented in *BMC Medicine *by Maitland *et al*. in this secondary analysis of the data in the FEAST trial have been keenly awaited and are of high importance [4].

### Gaining insight into the FEAST trial results: objectives of this study

The FEAST investigators considered that the increased mortality could be primarily due to refractory shock and/or the effects of fluid overload, particularly the development of pulmonary or cerebral edema. They considered that susceptibility to increased mortality would depend on the principal nature of the presenting syndrome (PS), characterized by features of severe shock or acidosis, hypoxic respiratory failure, or neurological causes such as seizures or coma. They defined terminal clinical events (TCE) at the time of death to characterize the principal cause of death according to cardiovascular, pulmonary or neurological criteria. This characterization of the PS and TCE using pragmatic, robust clinical criteria is unique and central to the face validity of this sub-study. The investigators also minimized bias by blinded adjudication of PS and TCE - essential for an unblinded study - and adhered to the principles of internal validity presented in the main paper with minimal loss to follow up, intention-to-treat analyses and no imputation for missing data.

Analyses were presented comparing bolus with no bolus as mortality was similar for both albumin and saline boluses, so elucidating differences in cause of death between the two fluids was not possible from these results.

### Results: cardiovascular collapse rather than fluid overload contributes most to excess deaths with rapid fluid resuscitation

Data are presented from a large cohort of patients (n = 2,396) who could be classified into a single or combination of PSs. Of these, the most common PS was severe shock or acidosis, either as a sole presentation or in combination with other PSs. It was these patients, particularly those with all three PSs, who had the highest mortality, suggesting that shock at presentation had a significant impact on mortality in both groups.

Of particular interest was the observation that there appeared to be a short-term clinical benefit, namely resolution of shock within one hour, in patients who received boluses. However, this effect did not translate into improved survival compared with those who did not receive boluses; nor was there a difference in mortality between patients who did and did not respond to fluid boluses.

When patients were categorized according to the TCE, the greatest difference in mortality was observed in patients who received boluses and who died from cardiovascular causes, specifically terminal arrhythmia or hypotension. This difference was greater in patients who died in the first hour. Contrary to expectations, there was no significant difference between patients who died primarily from respiratory or neurological causes, suggesting that fluid overload was not a predominant pathological mechanism.

The increased risk of death with boluses and the TCEs were similar in patients with or without malaria and with or without anemia. Concern that the results were restricted primarily to patients in low-income countries with malaria or severe anemia is not supported by these analyses. Consideration of cardiotoxic events of bolus fluid resuscitation in other critically ill patients, particularly those with severe sepsis, is therefore warranted.

### Interpretation: how do these results inform clinicians about potential pathological mechanisms associated with fluid bolus resuscitation?

Clearly, fluid resuscitation is only one component of a complex resuscitation strategy that is targeted at correcting deficits in circulating blood and plasma volume, intrinsic cardiac function and compensatory neurohormonal function. Under physiological conditions, the capacitant venous system has the potential to generate increases in venous return in response to hypovolaemia through sympathetically mediated responses - the 'stressed' volume [5,6].

The patients presented in this sub-study of the FEAST study had impaired perfusion rather than decompensated shock, which makes them a population of critically ill patients with maximal compensatory mechanisms. Although there was evidence of improved short-term hemodynamic effects with boluses in these patients, bolus administration may have resulted in a rapid reduction in sympathetically mediated compensatory mechanisms, leading to cardiovascular dysfunction and death.

An additional mechanism of increased cardiovascular causes of death may relate to treatment-induced hyperchloremic metabolic acidosis. When infused rapidly and in large volumes, albumin in saline carrier solutions may have the same effect on chloride and acid-base status as saline [7,8]. This effect is not seen when other crystalloid solutions with more physiological strong ion differences are infused [9].

The role of acidosis-induced cardiotoxicity remains uncertain, but this effect, in conjunction with a potential ischemia-reperfusion injury, may have been amplified in this patient population where close monitoring of acid-base status or support with mechanical ventilation or vasopressors was not possible.

However, this does not exclude the possibility that rapid high volume fluid resuscitation may be harmful under conditions with more resources. Trials of fluid resuscitation with colloids (albumin [10] and hydroxyethyl starch [11,12]) in high-income countries have also demonstrated transient improvements in indices of hemodynamic resuscitation, but these have not been associated with improvements in mortality or other patient-centered outcomes.

Furthermore, it appears that, as adjudicated by the TCEs, fluid overload was not a predominant cause of excess deaths in the patients in the FEAST study. This is of interest, particularly the low incidence of neurological causes of death. Although administration of albumin is associated with increased mortality in patients with traumatic brain injury [13,14], this was not evident in this patient population, even though a substantial proportion of patients had severe cerebral malaria.

Although it appears that bolus resuscitation with albumin and saline in critically ill children in resource-poor conditions cannot be recommended, further high-quality studies examining the effects of balanced salt solutions for fluid resuscitation are required in this patient population. Indeed, the question of the optimal resuscitation fluid, both the type and dose, remains uncertain in all critically ill patient population across all income regions.

## Conclusions

This sub-study of the FEAST study has provided unique insights into a critically important area of clinical medicine. Fluid resuscitation is under increasing scrutiny following the publication of a number of randomized controlled trials questioning the safety and efficacy of widely used resuscitation fluids. The importance of these findings make it imperative that clinicians carefully consider the type, dosing and rate of administration of resuscitation fluids and monitor their effects beyond the initial resuscitation period. Administration of resuscitation fluid requires as much thought and care as the administration of any other potentially lethal drug.

## Abbreviations

FEAST: Fluid Expansion as Supportive Therapy; PS: presenting syndrome; TCE: terminal clinical events.

## Competing interests

JM is supported by a Practitioner Fellowship from the National Health and Medical Research Council of Australia. His institution (the George Institute) has received unrestricted grant support, travel expenses and one speaker's fee to conduct the Crystalloid versus Hydroxyethyl Starch Study (CHEST) trial from Fresenius Kabi. SF's institution (the George Institute) has received unrestricted grant support and travel expenses to conduct the Crystalloid versus Hydroxyethyl Starch Study (CHEST) trial from Fresenius Kabi.

## Authors' contributions

JM and SF wrote the article and give final approval for the manuscript to be published.

## Authors' information

JM and SF are founding members and past-Chairmen of the Australian and New Zealand Intensive Care Society Clinical Trials Group. JM is past-President of the College of Intensive Care Medicine of Australia and New Zealand.

JM and SF hold professorial positions at the University of New South Wales and University of Sydney respectively. Through their involvement with the George Institute for Global Health, SF and JM were the Chief Investigators in two large-scale investigator-initiated randomized controlled trials of fluid resuscitation in intensive care patients: the Saline versus Albumin Evaluation (SAFE) and Crystalloid versus Hydroxyethyl Starch Trial (CHEST).

## Pre-publication history

The pre-publication history for this paper can be accessed here:

http://www.biomedcentral.com/1741-7015/11/67/prepub
